# Abatacept monotherapy compared with abatacept plus disease-modifying anti-rheumatic drugs in rheumatoid arthritis patients: data from the ORA registry

**DOI:** 10.1186/s13075-016-0956-7

**Published:** 2016-03-30

**Authors:** Marie-Elise Truchetet, Nicolas Poursac, Thomas Barnetche, Emilie Shipley, Jacques-Eric Gottenberg, Bernard Bannwarth, Christophe Richez, Thierry Schaeverbeke

**Affiliations:** Département de Rhumatologie, Hôpital Pellegrin, CHU de Bordeaux, Place Amélie Raba-Léon, 33076 Bordeaux, France; Groupe Epidemiologie Clinique des Maladies Inflammatoires d’Aquitaine (GECMIA) Study Group, CHU de Bordeaux, Place Amélie Raba-Léon, 33076 Bordeaux, France; Département de Rhumatologie, Hôpital de Dax, 18 Avenue Paul Doumer, 40100 Dax, France; Hôpitaux Universitaires de Strasbourg et Universités de Strasbourg, 1 Place de L Hôpital, 67000 Strasbourg, France

**Keywords:** Rheumatoid arthritis, Abatacept, Retention rate, Biologic agent, Monotherapy

## Abstract

**Background:**

Retention rate, efficacy, and safety of abatacept (ABA) was compared between patients with rheumatoid arthritis receiving ABA as monotherapy to those in combination ABA + conventional synthetic DMARD (csDMARD).

**Methods:**

The patients were obtained from the ORA registry. The retention rate was analysed in two ways: (1) therapeutic strategy retention, in which the addition of a csDMARD was considered to indicate failure of the monotherapy strategy; and (2) ABA retention, which was assessed by the discontinuation of ABA regardless of other treatment modifications. Efficacy and safety were compared between ABA initiated alone and ABA used in combination with a csDMARD.

**Results:**

The retention rate at month 6 (M6) was evaluated in 569 patients. A significant difference was identified in the retention rate between the ABA monotherapy strategy and the ABA + csDMARD strategy (58.5 % [110/188] vs. 68 % [258/381], respectively, *p* = 0.031). No significant difference was identified in the ABA retention rate initiated either as a monotherapy or in combination with csDMARDs (75 % [142/188] vs. 76 % [291/381], respectively, *p* = 0.824). Data regarding ABA efficacy were available for 444 patients. There was no significant difference in the responder proportion after 6 months of treatment between ABA monotherapy and ABA + csDMARD treatment (60.2 % [88/146] vs. 60 % [179/298], respectively, *p* = 0.967).

**Conclusions:**

This “real-life” analysis, which is relevant for bedside practice, emphasised the satisfactory efficacy and safety of ABA used in monotherapy, which provides an acceptable alternative when csDMARDs are undesirable.

## Background

In parallel with the discovery of new pathways and drugs in the pathogenesis of rheumatoid arthritis (RA), the development of new therapeutic strategies has been a key component in the improvement of RA care [1]. These strategies include the early introduction of disease-modifying anti-rheumatic drugs (DMARDs), treat to target and tight control [2, 3]. The latter gathers principles based on the rapid and sustainable control of inflammation by optimised treatments. The combination of methotrexate (MTX) and a biologic agent is considered as the standard strategy for RA that is responding poorly to conventional synthetic DMARDs (csDMARDs) alone. This has been recently disputed by an increasing number of studies, where combinations of synthetic DMARDs are non-inferior to combination biological DMARDs (bDMARDs) and synthetic DMARDs [4]. Since the earliest studies conducted with tumour necrosis factor alpha (TNF-α) inhibitors, it has been demonstrated that the therapeutic response to biological agents was improved by the addition of a csDMARD, primarily MTX [5, 6]. Subsequently, most clinical trials have evaluated bDMARDs in combination with MTX. However, MTX is sometimes contraindicated; it is also responsible for minor but bothersome side effects. Moreover, many patients do not understand why they must continue a treatment that has previously failed to improve their condition. As a consequence, all bDMARD registries have indicated that these agents are used in monotherapy in one-third of patients in daily practice [7]. Recently, the AMBITION and ACT-RAY studies discussed the equivalence of tocilizumab when used in monotherapy or in combination with MTX [8, 9].

Association with MTX is required by the French and European marketing authorisation for abatacept (ABA). Very few data are available regarding the use of ABA in intravenous monotherapy apart from the very recently published AVERT trial [10].

In the present study, we analysed data from the Orencia and Rheumatoid Arthritis (ORA) registry to compare ABA monotherapy with an ABA + csDMARD combined strategy, using the retention rate as the principal outcome. This study provides the first evaluation of the efficacy and safety of the ABA monotherapy strategy in ORA patients.

## Methods

### The ORA registry

The ORA registry is an ongoing, nationwide, prospective French cohort study that investigates the long-term safety and efficacy of ABA in the treatment of RA. It was established in June 2008 by the French Society of Rheumatology upon the drug’s approval for use in France [11]. The last patient in that study was included in the ORA registry in 2010. The ORA registry received approval from the French authorities and patients were included after written informed consent was obtained. Data at baseline, month 3 (M3) and month 6 (M6) were collected by trained clinical nurses or technicians in each centre.

### Population

#### Inclusion criteria

All patients from the ORA registry who had previously completed their 6-month follow-up visit were included in our study.

#### Exclusion criteria

A lower age limit of 18 years old was established, and patients with outliers among their data were excluded from the study. Patients who did not meet the 1987 American College of Rheumatology (ACR) criteria (primarily due to missing or inconsistent data in the registry) were excluded from the study.

#### Definitions of treatment groups

**Fig. 1:**
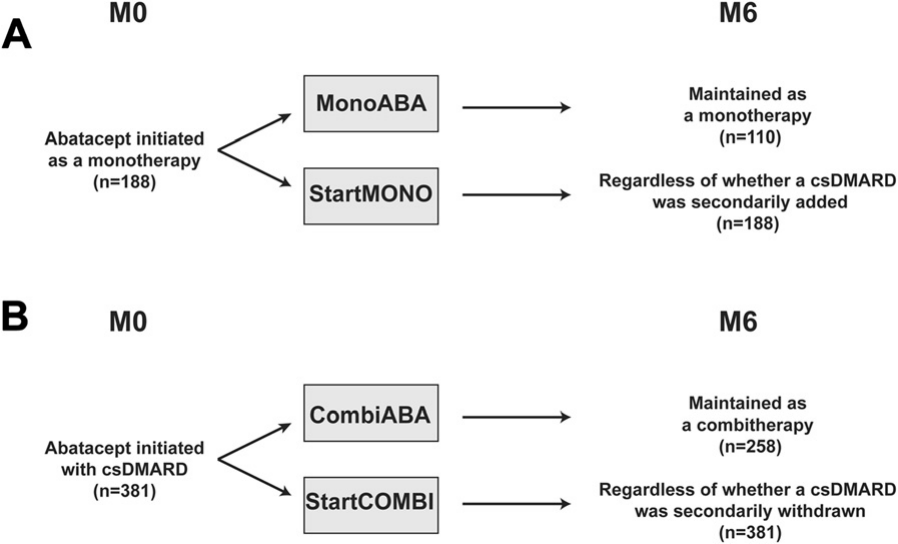
Groups of patients for retention rate calculations. Patients of the ORA registry were assigned retrospectively to two main groups. **a** Patients treated with ABA initiated as a monotherapy. In a first analysis, we considered patients for whom ABA was maintained as a monotherapy (MonoABA). In a second analysis, we considered all patients of this group regardless of whether a csDMARD was secondarily added (StartMONO). **b** Patients treated with ABA initiated in combination with a csDMARD. In a first analysis, we considered patients for whom ABA was maintained as a combination therapy with a csDMARD (CombiABA). In a second analysis, we considered all patients of this group regardless of whether the csDMARD was secondarily withdrawn (StartCOMBI). *ABA* abatacept, *csDMARD* conventional synthetic disease-modifying anti-rheumatic drug, *M0* month 0, *M6* month 6, *ORA* Orencia and Rheumatoid Arthritis

The patients were retrospectively assigned to two main groups according to the initiation of either ABA alone or ABA in combination with a csDMARD. In each group, two analyses were performed according to the initial treatment conditions (Fig. 1).

First, patients initially treated with ABA as a monotherapy, regardless of whether a csDMARD was secondarily added during the 6-month period of analysis were named StartMONO. A subset of this group continued ABA as a monotherapy throughout the 6-month period of analysis (MonoABA).

Second, patients initially treated with ABA in combination with a csDMARD, regardless of whether the csDMARD was continued or withdrawn during the 6-month period of analysis were named StartCOMBI. A subset of this group continued ABA in combination with a csDMARD throughout the 6-month period of analysis (CombiABA).

Patients in MonoABA and CombiABA are also included in StartMONO and StartCOMBI, respectively.

#### Assessment criteria and objectives

We assessed as the principal objective the retention of a treatment strategy (MonoABA vs. CombiABA) during the 6-month period, wherein a patient is treated in the same manner from the start to the end of follow-up.

Secondary objectives comprised the retention of ABA itself in StartMONO vs. StartCOMBI groups during the 6-month period observed. We also evaluated the efficacy in MonoABA vs. CombiABA and in StartMONO vs. StartCOMBI groups, assessed by the 28-item Disease Activity Score (DAS-28) erythrocyte sedimentation rate (ESR) score at month 0 (M0) and month 6 (M6). According to the European League Against Rheumatism (EULAR) criteria, treatment was considered effective when the EULAR response was good or moderate. The administration of corticosteroids was observed in the StartMONO and StartCOMBI groups. Finally, safety in the StartMONO and StartCOMBI groups, defined as the number of patients with at least one mild (clinical observation only without any intervention indicated), moderate (minimal intervention needed) or severe (hospitalization, and/or intravenous treatment required and/or resulting in death) adverse event during the 6-month period was assessed.

### Statistical analysis

Statistical analysis was performed using the STATA/SE software, version 13.1 (College Station, TX, USA: StataCorp LP). Appropriate testing was performed according to the results of normality tests. Student’s *t* test and the Mann-Whitney test (or Wilcoxon’s test for paired values) were used to analyse quantitative data, and the chi-square or Fisher’s exact test was used to analyse qualitative data. A *p* value <0.05 was considered statistically significant.

## Results

### Baseline demographics and characteristics of the population

**Fig. 2:**
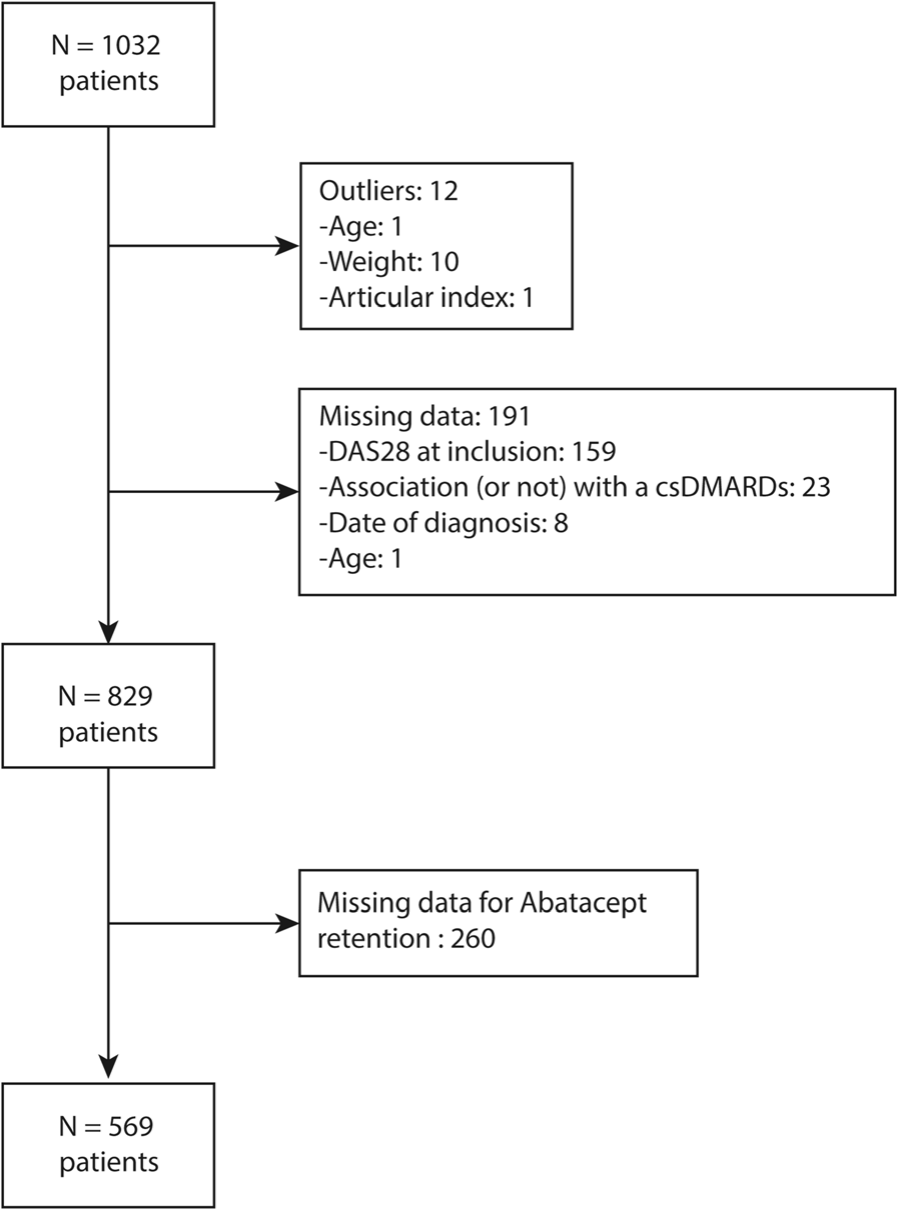
Flow chart illustrating the patient inclusion protocol for the study. Patients were excluded if their data consisted of outliers (n = 12) or contained errors in the collection process. Missing data constituted the main explanation for patient exclusion. At a minimum, treatment information at months 0 and 6 was required for inclusion

Of the 1032 patients included in the ORA registry, 829 (80.3 %) had been followed for at least 6 months at the time of analysis. Of these 829 patients, 276 (33.3 %) received ABA as a monotherapy at M0. A flow chart of the patient exclusion strategy is shown in Fig. 2. Exclusions were primarily due to missing data. The median age and disease duration were 60 (range: 20–89) and 14 (range: 2–51) years, respectively. The patients with fully available data for analysis were 90 % positive for anti-citrullinated protein antibodies (for a 70.9 % in the whole registry) [12]. ABA was administered as the first biological treatment in 12 % of the patients. One anti-TNF agent was used prior to ABA in 24 % of the patients, two agents in 40 % of the patients, and three agents in approximately 24 % of the patients. The clinical and biological characteristics were comparable between the StartMONO and StartCombi groups (Table 1).

**Table 1:** Clinical features of the 569 patients registered in the ORA and included in the present study

	Abatacept monotherapy (n = 188)	Abatacept + DMARDs (n = 381)
Sex, female n (%)	153 (81 %)	297 (78 %)
Age, median (range)	63 (22–89)	59 (23–89)
Duration of disease, median (range)	16 (3–51)	14 (2–45)
Inclusion DAS-28 score, median (range)	5.5 (2–8.9)	5.3 (1.6–8.5)
Previous anti-TNF therapy, n (%)		
0	26 (14 %)	38 (10 %)
1	47 (25 %)	85 (22 %)
2	72 (38 %)	156 (41 %)
3	43 (23 %)	102 (27 %)
Previous rituximab therapy, n (%)	72 (38 %)	104 (27 %)
Cortisone treatment, n (%)	144 (75 %)	280 (73 %)
Rheumatoid factor, n (%)	73/86 (85 %)	133/155 (86 %)
ACPA, n (%)	67/71 (94 %)	112/124 (90 %)
C-reactive protein, median (range)	14 (0.2–157)	12 (0–179)
Smoking, n (%)	18 (9 %)	49 (13 %)

*DMARD*s disease-modifying anti-rheumatic drugs, *DAS-28* 28-item Disease Activity Score, *TNF* tumour necrosis factor, *ACPA* anti-citrullinated peptide antibody

### ABA retention rate

Drug withdrawal or treatment modifications are indirect indicators of safety and efficacy and are fairly well represented by the retention rate [13]. No survival curve was performed because of too short a duration of follow-up (6 months) and of the small number of intermediate surveys. Of the 829 patients with complete follow-ups, data were fully available for 569 concerning the retention rate. At M0, 188 of these 569 patients initiated ABA as a monotherapy (StartMONO), and 381 patients initiated ABA in combination with a csDMARD (StartCOMBI).

#### MonoABA vs. CombiABA

In a first analysis, any changes in the ABA or csDMARD regimen were considered to represent a failure of the treatment strategy. We identified a significantly lower retention rate of the MonoABA group compared with the CombiABA group (58.5 % [110/188] vs. 68 % [258/381], *p* = 0.031). Seventy-eight patients failed to maintain abatacept in monotherapy, amongst whom 46 stopped the abatacept and 32 started a DMARD in complement. Furthermore, a significantly increased risk of strategy discontinuation was identified for the MonoABA group compared with the CombiABA group (relative risk [RR]: 1.48; 95 % confidence interval [CI]: 1.02–2.17).

#### StartMONO vs. StartCOMBI

In the second analysis, ABA retention rate was considered regardless of whether csDMARD treatment was added or stopped. We found that the ABA retention rate was similar in both the StartMONO and StartCOMBI groups (75 % [142/188] vs. 76 % [291/381], respectively, *p* = 0.824).

#### Reasons for ABA monotherapy discontinuation

We compared the reasons for ABA discontinuation in the entire sample population. The most common explanation for the termination of ABA treatment was primary ineffectiveness, with a comparable incidence in both the StartMONO and StartCOMBI groups (41.3 % in the StartMONO group vs. 44.4 % in the StartCOMBI group, *p* = 0.709). The distribution of other reasons for the discontinuation of ABA infusion was also comparable between the two groups and is shown in Table 2. Therapeutic escape was considered when primary efficacy was observed at M3 and not at M6.

**Table 2:** Reasons for discontinuation of abatacept infusion in the abatacept monotherapy group and the abatacept plus conventional DMARDs group

	Abatacept monotherapy (n = 46)	Abatacept + conventional DMARD (n = 90)
Primary ineffectiveness, n (%)	19 (41.3 %)	40 (44.4 %)
Therapeutic escape, n (%)	3 (6.6 %)	9 (10 %)
Infusion reaction, n (%)	2 (4.3 %)	1 (1.1 %)
Other side effects, n (%)	4 (8.7 %)	6 (6.7 %)
Unknown, n (%)	18 (39.1 %)	34 (37.8 %)

*DMARD*s disease-modifying anti-rheumatic drugs

### Efficacy

#### MonoABA vs. CombiABA

In the MonoABA (n = 110) and CombiABA (n = 258) groups, data for the evaluation of the EULAR response were available for 99 and 223 patients, respectively. The efficacy of these strategies at M6 was similar in both groups, with 70.7 % (70/99) good or moderate responders in the MonoABA group vs. 67.7 % (151/223) in the CombiABA group (*p* = 0.592).

#### StartMONO vs. StartCOMBI

The necessary data to determine efficacy were available for 444 patients. The efficacy of ABA at M6 was similar in both groups, with approximately 60 % good or moderate EULAR responses in the treated patients: 60.2 % (88/146) in the StartMONO group vs. 60 % (179/298) in the StartCOMBI group (*p* = 0.967).

### Corticosteroids

Corticosteroids were frequently co-administered with ABA in RA in general and in the ORA registry in particular. Increases in corticosteroid dosage could reflect the poor effectiveness of DMARDs, whereas corticosteroid tapering or withdrawal might attest to DMARD efficacy. Data regarding the corticosteroid doses at M0 and M6 were available for 323 patients. The frequency of patients who required an increase in corticosteroid dose was equivalent between the StartMONO and StartCOMBI groups (10/98 patients (10.2 %) vs. 28/225 patients (12.4 %), respectively, *p* = 0.566). In addition, an equivalent reduction in the corticosteroid dose was identified in both groups (-3.31 ± 7.94 in StartMONO and -2.44 ± 5.59 in StartCOMBI, *p* = 0.25).

### Safety

In terms of safety, the main reason to discontinue ABA treatment was the occurrence of infusion reactions. There were no significant differences between the StartMONO and StartCOMBI groups regarding the incidence of infusion reactions (4.3 % vs. 1.1 %, respectively, *p* = 0.22) or frequency of other side effects (8.7 % vs. 6.7 %, respectively, *p* = 0.67).

In terms of serious adverse events, the incidence of cancers was not significantly different between the StartMONO and StartCOMBI groups (3.7 % vs. 3.4 %, *p* = 0.850), and there was no predominance of a particular type of cancer.

The global incidence of infections was not significantly different between the groups (12.2 % vs. 10.7 %, *p* = 0.0601). Unfortunately, we could not distinguish these events as minor or severe infections because of a lack of precision in the collected data.

Liver function tests were available for 438 patients. No significant difference was identified between the StartMONO and StartCOMBI groups in terms of liver abnormalities (18.9 % [29/153] vs. 14 % [40/285], respectively, *p* = 0.178).

## Discussion

Based on the analysis of ORA registry data, this study demonstrated that the ABA monotherapy strategy had a lower retention rate compared with the combination strategy, with an estimated relative risk of failure of 1.48. In addition, the ABA molecule had a similar retention rate at 6 months, regardless of whether the ABA treatment was initiated as a monotherapy or in combination with a csDMARD. Finally, efficacy and safety were comparable amongst all treatment groups, even if we have no information on efficacy in the subgroup of StartMONO patients who secondarily initiated a DMARD. The results suggested that the combination strategy was more successful than the monotherapy strategy in term of maintenance. However those that were successful on monotherapy showed equivalent efficacy, whilst in those requiring treatment adjustments, comparable retention and response could be achieved.

The use of registries offers the great benefit of analysing real-life data from non-selected patients, without the constraints of physician prescriptions. However, our study had some limitations. One major limitation was the short follow-up period, which was restricted to 6 months. This follow-up time is too short for a survival analysis. Six months is not a long enough observational period to draw definitive conclusions about the drug survival. Nevertheless, this period of follow-up corresponds to daily practice: often, a 6-month period is routinely necessary to assess a DMARD’s efficacy. Nevertheless, a long-term analysis of the ORA data would be interesting to complete our study. We chose the treatment retention rate as the main criterion for the global assessment of efficacy, because of the frequently missing data for the calculation of the DAS-28. A drug maintenance rate reflects the effectiveness, safety, acceptance and tolerability of a medication, and it appears to be a good criterion for drug evaluation in daily practice. Another limitation implied by this short follow-up time was that we could not consider the structural progression within the 6-month period. Our study is limited by the high number of patients with missing data of ABA retention is very high. The lack of data is explained by the number of patients who did not complete the 6-month visit at the time of the end of our study and by the missing data due to patients lost to follow-up.

These caveats are usually found in registry observational studies.

The monotherapy issue is of great relevance, as one-third of patients are treated with monotherapy in daily practice and registries, regardless of the reason, i.e. real contraindications for synthetic DMARDs, patient preference or compliance [5–7]. In addition, some patients do not understand why they are maintained on treatments that previously failed to control their disease [14]. A definitive discontinuation of MTX because of intolerance was reported in 10.5 % of treated patients in a systematic analysis of the literature [15].

Nonetheless, clinical trials have established that the therapeutic effects of most biological agents are superior when used in combination with MTX rather than as monotherapies [16]. Efficacy of combined treatments (csDMARD + bDMARD) and even superiority of the combination over monotherapy was demonstrated very early for TNF-α blockers [5–10, 17–23]. The results have been more equivocal for tocilizumab monotherapy. The AMBITION and ADACTA trials indicated the superior efficacy of tocilizumab over MTX and adalimumab monotherapies, respectively [8, 11, 24]. In the ACT-RAY trial, a comparison of tocilizumab + MTX with tocilizumab alone in RA patients with an inadequate response to MTX found no significant difference between the groups in clinical efficacy at week 52 [9]. However, a difference was identified in the percentage of patients with non-significant radiographic progression: 92.8 % in the combination group vs. 86.1 % in the tocilizumab monotherapy group (*p* = 0.016) [25].

Very few data are available regarding ABA monotherapy. Immunogenicity of ABA has been shown to be very low [26]. The ACCOMPANY open-label trial assessed the immunogenicity, safety and efficacy of subcutaneous ABA administered with or without MTX [27]. The main result of that study was the weak immunogenicity of ABA, either as a monotherapy or in combination with MTX, wherein few patients developed anti-drug antibodies; these findings suggest that monotherapy could be an interesting option for this molecule. No differences were identified between the groups in clinical efficacy or safety. The AVERT trial compared ABA, ABA + MTX or MTX in early and severe RA patients. The combination of ABA + MTX demonstrated greater efficacy than either MTX or ABA alone, with 60.9 % of patients achieving DAS-28 remission at 1 year in the ABA + MTX group compared with 45.2 % and 42.5 % in the MTX and ABA monotherapy groups, respectively. Safety was comparable amongst all treatment groups [10]. Thus, these two trials led to divergent results, which might be explained by important differences in methodology: an open-label trial vs. a randomised, double-blind study, as well as different patient sample sizes and inclusion criteria.

These results are reinforced by a recent network meta-analysis that evaluated the comparative effects of biologics as monotherapies and biologics in combination with MTX on patient-reported outcomes (PROs) [28]. The meta-analysis concluded that, in patients who inadequately respond to csDMARDs, the efficacies of a TNF-α inhibitor, ABA, and tocilizumab combined with MTX were comparable. In contrast, tocilizumab as a monotherapy was associated with an increased improvement in pain and PROs compared with TNF-α inhibitors. ABA as a monotherapy was not evaluated in the meta-analysis.

Data obtained from clinical trials do not reflect daily life for many reasons, including the selection criteria for patients and the lack of adaptation of the treatment according to patient response. In the STURE registry, it was noted as early as 2003 that patients treated with etanercept + MTX obtained a significantly lower DAS-28 score than patients who received etanercept alone [29]. In the BSR biologic registry, a better response to TNF-α inhibitors was associated with the concomitant use of MTX, although statistical significance was only reached for etanercept (odds ratio [OR] = 1.82, 95 CI: 1.38–2.40) [30]. In the DANBIO registry, the combination of MTX with TNF-α inhibitors was associated with fewer drug withdrawals and a greater proportion of good EULAR responses [31]. Data from the same Danish registry was recently analysed for monotherapy use of biologics with very little information on ABA. The overall conclusion was that one biologic agent in five was used in monotherapy with a remission rate and drug adherence equivalent except for infliximab. No direct comparison with combination with csDMARD was made [32]. In a retrospective analysis of a cohort of patients who were first administered a TNF-α inhibitor, the switching rate was significantly increased when the drug was taken as a monotherapy than when it was used in combination with MTX [33]. In a Japanese registry, an increased incidence of discontinuation because of insufficient efficacy was identified in patients who received etanercept treatment without concomitant MTX (hazard ratio [HR] = 2.226, 95 % CI 1.363–3.634) [34].

For non-TNF-related biologics, to our knowledge, there has been only one real-life report of an analysis of the effect of MTX co-administration, which concerned both tocilizumab and ABA [35]. In that study, concomitant MTX was not predictive of a better response for either tocilizumab or ABA. However, only approximately 50 % of the patients used concomitant MTX in the Danish registry, and there were no precise data regarding the persistence of MTX combination over the study period.

## Conclusions

In conclusion, our study suggested that the combination strategy was more successful than the monotherapy strategy in term of retention. However, patients successful on monotherapy showed equivalent efficacy, whilst in those requiring treatment adjustments, comparable retention and response could be achieved. The lack of structural data and the short follow-up temper our conclusions, and this topic should be explored in an analysis involving a longer period.

## Abbreviations

ABA: Abatacept
ACR: American College of Rheumatology
bDMARD: Biological disease-modifying anti-rheumatic drug
CI: Confidence interval
csDMARD: Conventional synthetic disease-modifying anti-rheumatic drug
DAS-28: 28-item Disease Activity Score
DMARD: Disease-modifying anti-rheumatic drug
ESR: Erythrocyte sedimentation rate
EULAR: European League Against Rheumatism
HR: Hazard ratio
M0/M6: Month 0/month 6
MTX: Methotrexate
OR: Odds ratio
ORA: Orentia and Rheumatoid Arthritis
PROs: Patient-reported outcomes
RA: Rheumatoid arthritis
RR: Relative risk
TNF: Tumour necrosis factor
